# Manufacturing of Sustainable Untreated Coal Ash Masonry Units for Structural Applications

**DOI:** 10.3390/ma15114003

**Published:** 2022-06-04

**Authors:** Wasim Abbass, Safeer Abbas, Fahid Aslam, Ali Ahmed, Tauqir Ahmed, Agha Hashir, Amr Mamdouh

**Affiliations:** 1 Department of Civil Engineering, University of Engineering and Technology, Lahore 54890, Pakistan; wabbass@uet.edu.pk (W.A.); safeerabbas26@gmail.com (S.A.); ali@uet.edu.pk (A.A.); hashiraghakhan@hotmail.com (A.H.); 2 Department of Civil Engineering, College of Engineering in Al-Kharj, Prince Sattam Bin Abdulaziz University, Al-Kharj 16273, Saudi Arabia; 3 Department of Civil Engineering, National University of Computer and Emerging Sciences, Lahore 54000, Pakistan; tauqir.ahmed@nu.edu.pk; 4 Architectural Engineering Department, Faculty of Engineering and Technology, Future University in Egypt, New Cairo 11835, Egypt; amr.mamdouh@fue.edu.eg

**Keywords:** sustainable construction materials, efficient waste disposal, recycling of coal ash

## Abstract

Burnt clay bricks are one of the most important building units worldwide, are easy and cheap to make, and are readily available. However, the utilization of fertile clay in the production of burnt clay bricks is also one of the causes of environmental pollution because of the emission of greenhouse gases from industrial kilns during the large-scale burning process. Therefore, there is a need to develop a new class of building units (bricks) incorporating recycled industrial waste, leading toward sustainable construction by a reduction in the environmental overburden. This research aimed to explore the potential of untreated coal ash for the manufacturing of building units (coal ash unburnt bricks). Coal ash unburnt bricks were manufactured at an industrial brick plant by applying a pre-form pressure of 3 MPa and later curing them via water sprinkling in a control shed. Various proportions of coal ash (i.e., 30, 35, 40, 45, 50, and 55%) were employed to investigate the mechanical and durability-related properties of the resulting bricks, then they were compared with conventional burnt clay bricks. Compressive strength, flexural strength, an initial rate of water absorption, efflorescence, microstructural analysis via scanning electron microscopy, and cost analysis were conducted. The results of the compressive strength tests revealed that the compressive strength of coal ash unburnt brick decreased with an increase in the content of coal ash; however, up to a 45% proportion of coal ash, the minimum required compressive strength specified by ASTM C62 and local building codes was satisfied. Furthermore, bricks incorporating up to 45% of coal ash also satisfied the ASTM C62 requirements for water absorption. Coal ash unburnt bricks are lighter in weight owing to their porous developed microstructure. The cost analysis showed that the utilization of untreated, locally available coal ash in brick production leads us on the path toward more economical and sustainable building units.

## 1. Introduction

Burnt clay bricks comprise one of the most important building units in the construction industry. Around 20,000 brick kilns (mostly in a Bull’s Trench layout) are found in Pakistan alone for the production of burnt clay bricks, causing serious environmental issues (i.e., air pollution and the consumption of fertile clay) [1]. Approximately 1.4 trillion units of fired clay bricks are manufactured worldwide, and 340 billion tons of clay have been excavated around the globe for the production of clay bricks [2]. However, their quality and strength characteristics are dependent on several variables, including their constituents, such as clay, water content, and curing before burning [3]. Furthermore, the firing temperature in the kiln plays a vital role in achieving the brick’s desired properties. This causes a serious threat to the environment and leads to an increase in the initial cost of manufacture due to costly firing processes [4]. Therefore, an alternative to conventional burnt clay bricks is required for sustainable and pollution-free construction. Mega-scale infrastructure development (i.e., thermal coal-power plants) in the energy sector leads to the generation of a huge amount of industrial waste. Hence, around 730 million tons of industrial waste (i.e., coal ash) is produced and disposed of in open landfills, causing an environmental overburden that may eventually threaten human life [5]. Therefore, the efficient reuse of untreated coal ash generated from local coal power plants can be an important component in the sustainable management and disposal of waste.

Coal ash generated by thermal power plants is largely consumed in the construction industry, in various countries such as China, India, the United States (US), and the European Union (EU), among others. It is estimated that around 86%, 66%, 51%, and 35% of coal ash is used in the construction industries of China, India, the US, and the EU, respectively [6]. A great deal of published research is available on the utilization of coal ash/fly ash in the construction industry, especially in concrete [7,8,9]. The most common areas of applications of coal ash in construction include the production of high-strength concrete [10], as a base material for roads [11], waste stabilization [12], clinker formation [13], and, recently, in the production of geopolymer concrete [14], bricks, and blocks [15,16]. The utilization of coal ash for the production of new building units with reliable and quantifiable mechanical properties offers a positive impact on the environment by conservation of natural resources used for brick manufacturing [17]. Furthermore, coal ash is also a multifaceted material, containing around 188 mineral groups and 316 individual minerals [18]. Metallic oxides were the major constituents with unburnt carbon contents. Usually, the principal oxides are in the following order: SiO_2_ > Al_2_O_3_ > Fe_2_O_3_ > CaO > MgO > K_2_O [17]. One of the major areas for the consumption of coal ash in construction industries is in the production of bricks incorporating coal ash.

In South Asia, Pakistan is one of the largest brick producers. About 45 billion bricks are manufactured in Pakistan according to Business Record 2017 [19]. Conventional burnt clay brick production involves the utilization of the top layer of fertile soil and the burning of the clay beneath, leading to serious environmental concerns due to the hazardous emissions produced by brick kilns [20]. In recent years, sustainable bricks utilizing waste material have been manufactured [21,22,23,24]. Furthermore, the production of unburnt compressible waste bricks involving different parameters (i.e., the type of waste material, optimization of the constituents, and curing temperature) are reported elsewhere [25,26,27]. However, there is very scant published literature available [28] regarding the utilization of untreated coal ash (CA) for the production of sustainable compressible bricks. Therefore, an evaluation of the developments in unburnt compressible waste brick using untreated coal ash needs to take place for sustainable brick manufacturing. A sustainable compressed brick utilizing coal ash (as an alternative to burnt clay bricks) has been investigated in the current paper, working toward environmental protection by reducing CO_2_ emissions and saving fertile land. This research aimed to replace 100% of clay with waste coal ash and ordinary Portland cement for the production of bricks on a massive scale. The production of coal ash bricks will eliminate the costly firing process of conventional clay bricks, leading to more economical and sustainable construction.

Various researchers have studied the use of processed coal ash/fly ash for the production of ash bricks [6,15,21]. However, the uses of untreated raw coal ash that is directly collected from the deposition sites in construction activities are limited, because of its inferior properties compared to conventional fly ash. Therefore, this study aims to investigate the suitability of untreated coal ash as a potential material for the production of eco-friendly compressed bricks.

Different quantities of coal ash and cement were investigated to evaluate the optimum percentages for the production of water-cured compressible bricks. The research program was executed in three phases. In the first phase, the material characterization of raw untreated coal ash, cement, and quarry dust was performed using micro-structural (scanning electron microscope (SEM)) and energy dispersive X-ray diffraction (EDX) analysis. In the second phase, water-cured compressible bricks were manufactured and tested for their mechanical and durability properties. In the third phase, cost analysis for sustainable coal ash and conventional burnt clay bricks was evaluated for practical application purposes. Hence, the current research program provides insight and knowledge for improving water-cured compressible bricks. Furthermore, this research work contributes to their numerous social and economic benefits by utilizing waste material in the resulting value-added product. The main scope and aims of this research program were to analyze the suitability of untreated coal ash as an alternative to conventional construction material. Furthermore, this research leads to the development of sustainable, eco-friendly, and economical solutions for building low-cost housing projects.

## 2. Materials and Methods

### 2.1. Materials

**Figure 1:**
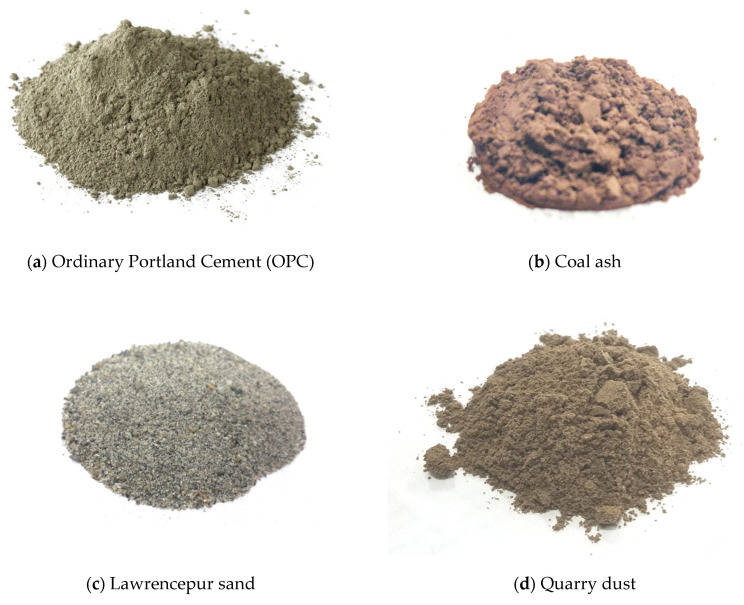
Physical appearance of the materials.

Ordinary Portland Cement (OPC), coal ash, fine sand, and quarry dust were used in the experiments. Locally available OPC and untreated raw coal ash from a thermal power plant were used in the current research, as shown in Figure 1a,b. Fine sand from Lawrencepur was procured, then quarry dust from manufacturing Marghalla crush was used for the production of brick, as shown in Figure 1c,d. The physical and chemical properties of the above-mentioned materials are summarized in Table 1.

### 2.2. Methodology

The research work was carried out in three phases. In the first phase, the material characterization of the raw material used in the current research was carried out. A scanning electron microscope (SEM) attached to an energy dispersive X-ray (EDX) was used for the microstructural analysis of cement, coal ash, sand, and quarry dust. Moreover, X-ray diffraction (XRD) analysis of the coal ash, sand, and quarry dust was carried out. Various chemical and physical properties of the raw materials were elucidated for the potential use of these materials in the development of water-cured compressible bricks. The coal ash and quarry dust were sieved before use through sieve numbers 200 and 50, respectively. In the second phase, water-cured compressible coal ash bricks incorporating different quantities of CA and cement were manufactured in a brick manufacturing plant and were then tested for their mechanical (compressive and flexural strengths) and durability properties (water absorption, the initial rate of absorption, efflorescence) for the mixtures defined in Table 2 in accordance with ASTM C67. In the third phase, a cost analysis for sustainable coal ash and conventional burnt clay bricks was evaluated for practical application purposes. Compressive strength and flexural strength were investigated at 28, 56, 90, and 120 days using 8 similar brick specimens for each time point. The weight per unit area was evaluated at 28 and 56 days, using 8 similar brick specimens. Water absorption and efflorescence were studied at the 28-day point, using 5 similar brick specimens. Around 100 brick specimens were prepared for every single proportion of coal ash, and around 600 bricks were manufactured for complete testing. The chemical changes and microstructural behavior in the tested brick specimens were studied through SEM and EDX analysis.

### 2.3. Casting of Brick Specimen

**Figure 2:**
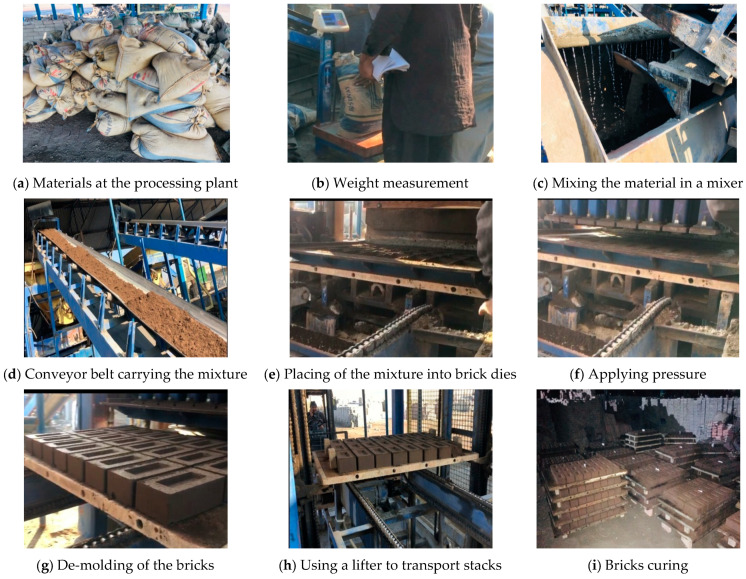
The casting process of coal-ash compressed bricks.

Coal-ash compressed bricks, sized 228 × 114 × 75 mm, were manufactured at an industrial brick manufacturing plant (Figure 2). First of all, raw materials (coal ash, cement, sand, and quarry dust) were weighed according to the desired mixtures (Table 2). All raw materials were then transferred to a one-ton capacity mixer (Figure 2c). The water-to-cement ratio was 0.15. Initially, dry mixing was performed for 3 min. Afterward, water was added piecewise to attain uniform mixing. Mixing was continued for 5 to 8 min. For each batch, 24 bricks were cast. In total, more than 200 bricks were required for conducting all the above-mentioned tests; therefore, 10 batches were cast for each mixture. Each batch of the prepared mixture was transferred to brick dies (molds) using a conveyor belt (Figure 2d,e).

After filling the brick molds with the mixture, a pressure of 3 MPa was applied to each specimen, using an automatic jack system attached to the brick-casting machine. The pressure was applied for 3 s. The bricks were then taken out from their respective molds (Figure 2g) and transported to the curing room (Figure 2h,i). The relative humidity and temperature in the curing room were >95% and 23 °C, respectively. Brick specimens were removed from the curing room after 28 days and underwent various tests.

## 3. Results and Discussion

### 3.1. Material Characterization

**Figure 3:**
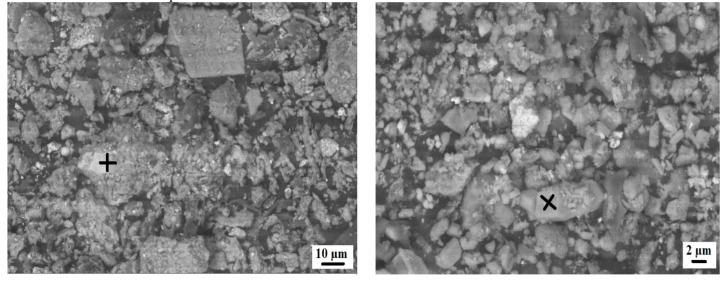
Microstructure of untreated coal ash.

**Figure 4:**
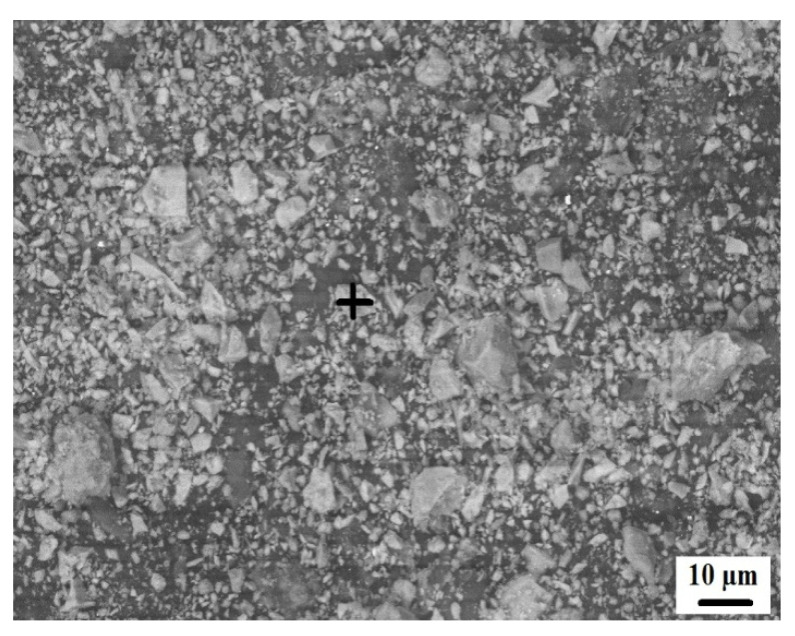
Microstructure of quarry dust.

**Figure 5:**
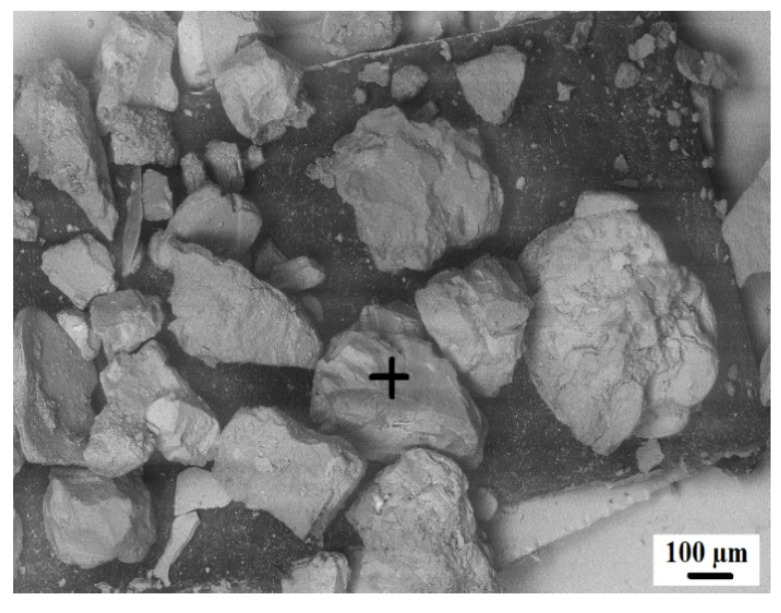
Microstructure of fine aggregate.

**Figure 6:**
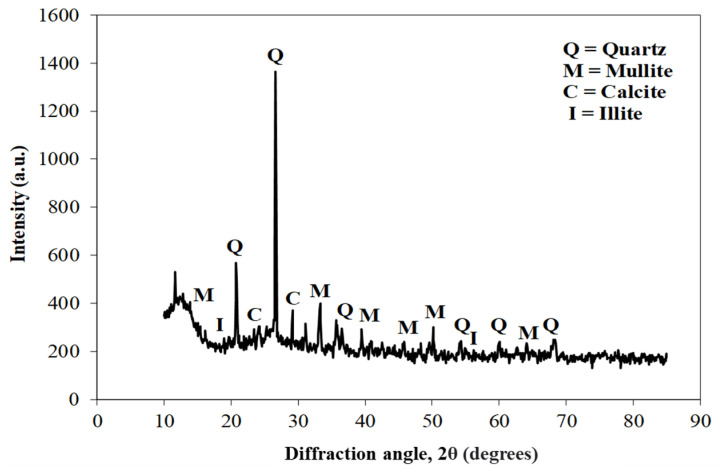
XRD of coal ash.

**Figure 7:**
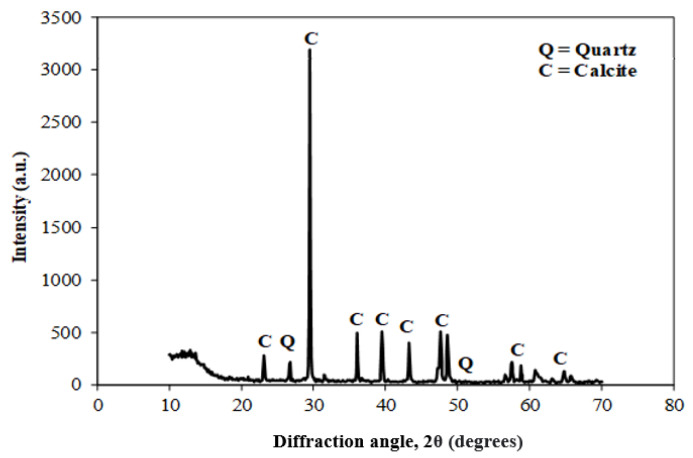
XRD of quarry dust.

**Figure 8:**
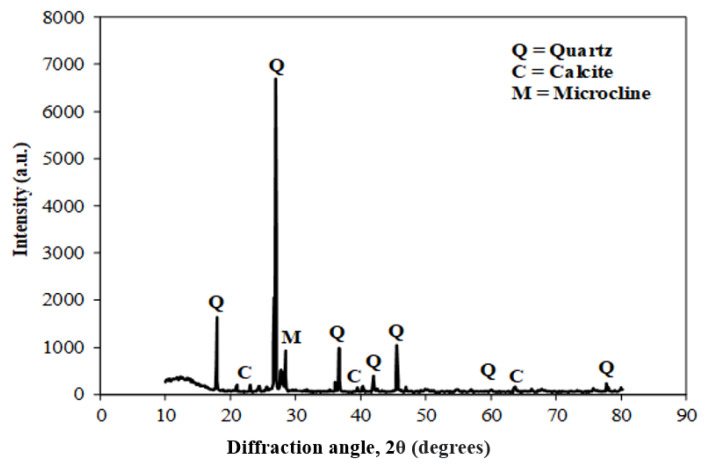
XRD of Lawrencepur sand.

Figure 3 shows the scanning electron microscope (SEM) images of coal ash (CA). It was observed that the CA particles were of varying sizes and irregular shapes. The SEM image (Figure 4) of quarry dust showed the presence of various sizes of particles with varying shapes. Figure 5 showed the SEM image of sand. The sand grains were observed to comprise irregularly shaped particles. The CA, quarry dust, and sand were examined using X-ray diffraction (XRD) to identify the crystalline components (Figure 6, Figure 7 and Figure 8). The components present in CA constituted quartz, mullite, calcite, and illite. Similarly, the XRD patterns of quarry dust and sand (Figure 7 and Figure 8) showed the presence of both quartz and calcite.

Table 1 also shows the chemical composition of the materials (i.e., coal ash, cement, and quarry dust) used in the production of the bricks. The main constituents of the cement were calcium oxide (61.47%), silica oxide (19.06%), aluminum oxide (5.68%), and iron oxide (4.34%) with other alkalis, with a loss on ignition of 3.24%. The coal ash was composed of oxides of silica (61.5%), alumina (10.3%), and calcium oxide (6.08%). The total sum of the silica and alumina in the coal ash was greater than 70% and can be classified as Class F fly ash, as per ASTM C618 (ASTM C618, 2019). Higher amounts of oxides in the coal ash from power plants were in agreement with previous studies [28,29]. Various other limits of ASTM C618 were also satisfied. For instance, a percentage of CaO is also less than 18%, as specified in the ASTM standards. The loss on ignition (LOI) of used coal ash was 10.39. A greater amount of loss on ignition in the coal ash has already been reported in other studies [30,31]. The higher loss on ignition in coal ash will result in a higher demand for water for targeted workability, leading to decreased compressive strength due to the unburnt elements [32]. Moreover, a higher LOI will lead to discoloration of the brick specimen. The chemical composition of sand used for the production of brick indicates that it has a greater amount of silica oxide (91.7%), with a lesser LOI (1.94%). The quarry dust was composed of oxides of calcium (42.84%), silica (9.7%), magnesium (2%), and aluminum (1.72%), with an LOI of 38.41%, which indicates the higher number of unburnt particles that are present in quarry dust.

### 3.2. Mechanical and Durability Properties of Bricks

#### 3.2.1. Compressive Strength

**Figure 9:**
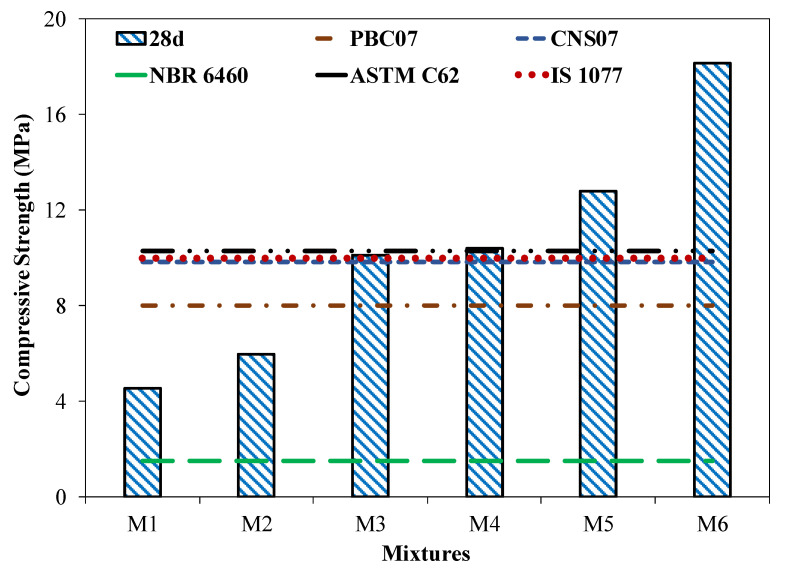
Compressive strength of the brick specimen with different CA contents.

Figure 9 shows the results of the compressive strength tests of coal ash bricks with various quantities of coal ash and cement content at 28 days. The results presented show an average of five specimens with a coefficient of variation (Table 3) of less than 5%. The compressive strength of coal ash brick was in the range of 4.5 to 18.1 MPa. It was observed that the compressive strength of the brick specimen increased with an increase in cement content. For example, the maximum compressive strength of 18 MPa was achieved by the brick specimen with a 30% addition of coal ash and 30% cement content (M6 mixture) in comparison to 4.5 MPa for the brick specimen that incorporated 55% coal ash and 5% cement content (M1 mixture). An increase in the compressive strength of the brick specimen was within the range of 30–300% for bricks incorporating a coal ash content of 30 to 55%. This increase in compressive strength from the addition of cement and coal ash was mainly attributed to the formation of calcium silicate hydrate (C-S-H) gel (hydration products), formed due to the hydration process [33]. More C-S-H gel was formed with an increase in the amount of cement, in combination with the coal ash present in the bricks, leading to increased compressive strength. For instance, mixture M6, which has a 30% cement content, has higher compressive strength compared to that of the M1 mixture incorporating a 5% cement content. Furthermore, secondary calcium silicate hydrate will form when the remaining calcium hydroxide reacts with the coal ash in the mixture; it tends to increase the compressive strength of the mixture [33]. Similar results were observed in the previous study [34].

Moreover, the compressive strength behavior of coal ash brick specimens incorporating different quantities of cement content was compared with ASTM C62-13a, 2013 [35], Chinese National Standard CNS 382 [36], Brazilian Standard NBR 6460 (ABNT 1983a) [37], Pakistan Building Code 2007 [38], Indian Standard (IS) 1077, 2007 [39] and ASTM C55-2017 [40]. It was observed that, beyond a 15% addition of cement in the brick manufacture, a combination with 45% coal ash (M3, M4, M5, and M6 mixtures) satisfied the standards specifications (CNS 382, PBC 07, NBR 6460, IS 1077, ASTM C62). Indian and Chinese standards were used for the compressive strength comparisons because Pakistan has very similar weathering, climatic, construction, and domestic conditions as those in both countries. The universally accepted ASTM standard was also chosen for comparison.

Table 4 summarizes the range of compressive strengths specified by the different standards used. ASTM C62 categorizes brick into three weathering conditions from severe to negligible, with a minimum compressive strength of 10.3 MPa for negligible weathering conditions. Similarly, the results for coal ash bricks were also compared with a concrete masonry brick unit, in accordance with ASTM C55. The Building Code of Pakistan classified 8 MPa as being the minimum compressive strength required for brick masonry. It was evident that 45% coal ash, along with a 15% addition of cement (M3 mixture) satisfied the PBC and ASTM limits for negligible weathering. The Chinese National Standard classifies bricks as first class and second class, with compressive strengths of 15 MPa and 9.8 MPa, respectively. In comparison to this Chinese standard, the coal ash brick with 45% coal ash content, incorporating 15% cement (M3 mixture), fell into the category of a second-class brick. In the Indian standards, bricks have been categorized into two types, i.e., load-bearing and non-load-bearing. It was observed that the brick specimen with a 5 to 10% addition of cement, in combination with a percentage of coal ash of 55 to 50%, fell into the category of the non-load bearing range of compressive strength, whereas the brick specimen incorporating 15% cement and above satisfied the load-bearing requirements of bricks, according to the Indian standard. Therefore, bricks manufactured with mixtures M3, M4, M5, and M6 can be used for load-bearing structural applications in compliance with the Indian building standards. Finally, the brick specimens were compared with the Brazilian building standards, which sets a limit of 1.5 MPa. All the brick specimens incorporating 30 to 55% coal ash (M1, M2, M3, M4, M5, and M6) satisfied the minimum specified limits of compressive strength.

#### 3.2.2. Effect of Curing Age on Compressive Strength

**Figure 10:**
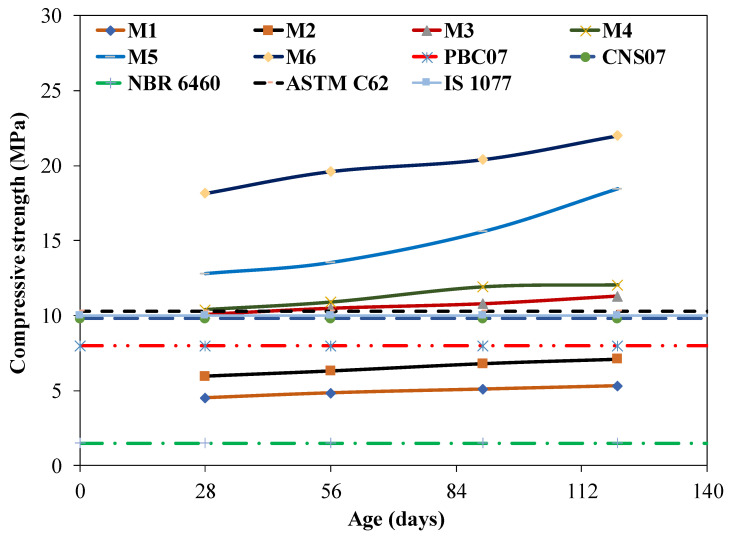
Compressive strength according to curing time.

Figure 10 shows the variations in compressive strength in brick specimens with different curing times for the different contents of coal ash incorporating the various cement contents. The average of five specimens is presented with a coefficient of variation (COV) that was less than 5%. It can be observed that the compressive strength of the bricks increased with the increased curing time. For instance, the compressive strength of the M6 mixture was 18 MPa at 28 days, compared to that of 22 MPa at 120 days. Moreover, bricks incorporating a lower coal ash content have a higher rate of gain in compressive strength compared to that of bricks incorporating a higher coal ash content. For instance, 55 to 40% coal ash in addition to the cement content has shown a maximum of 15% gain in strength from 28 days to 120 days. Conversely, 45 to 30% coal ash, in addition to the cement content, has shown a maximum 40% gain in strength at 120 days. The rate of gaining in strength with an increase in cement content may be because the increase in cement content leads to the formation of more CSH gel, leading to a significant pozzolanic reaction after 28 days [34]. Most brick samples gained strength up until 90 days with proper curing for the development of the compressive strength of the brick specimen.

#### 3.2.3. Modulus of Rupture

**Figure 11:**
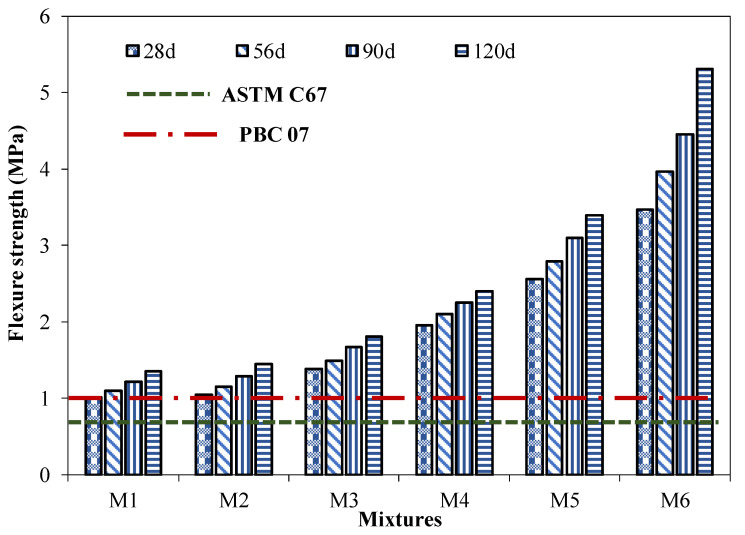
Flexural strength of the brick specimens with age.

The modulus of rupture of the different brick specimens with various quantities of coal ash and cement content is shown in Figure 11, with the average results of five specimens. The COV of all the tested specimens was below 4%. The results revealed that the flexural strength of the brick specimen decreased with the increase in coal ash content. Meanwhile, bricks with a lower content of coal ash and an increased amount of binding agent have a higher value for the modulus of rupture (MOR). The MOR of brick specimens increased from 1.01 MPa to 3.47 MPa for a 55 to 30% decrease in coal ash content at 28 days. It may be attributed to an increase in CSH gel formation due to an increased amount of pozzolanic reaction [33]. Similar findings were reported in the previous study [34].

An increase in MOR with curing time was observed for all the brick specimens due to the continuous hydration process, leading to the formation of more CSH gel [34]. The minimum MOR of the brick specimen was observed to be 1.01 MPa for 55% coal ash content, along with a 5% cement content. However, the flexural strength shown by the bricks with the minimum content of cement satisfied the requirements of the Pakistan Building Code 2007 [38] and ASTM C67 [41].

#### 3.2.4. Weight per Unit Area

**Figure 12:**
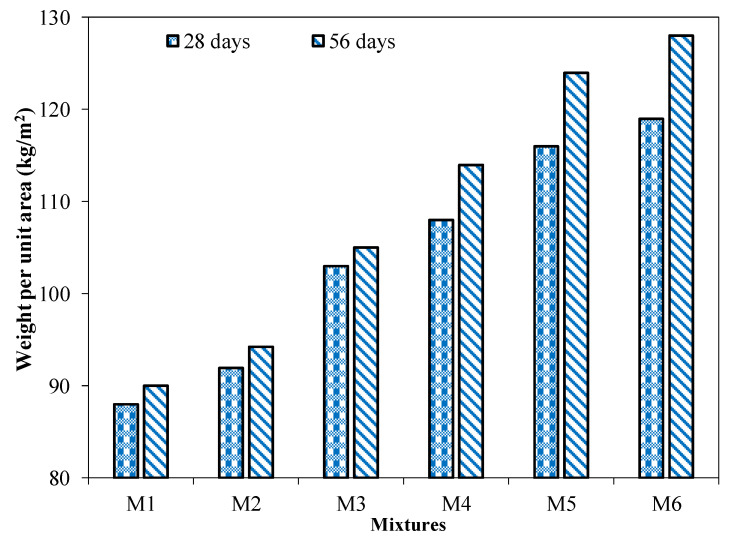
Weight per unit area of the brick specimen with different quantities of cement.

Figure 12 shows the weight per unit area for unburnt bricks incorporating different contents of cement and coal ash. The results revealed that the weight per unit area of brick specimens incorporating different coal ash contents increased with the decrease in the percentage of coal ash. For instance, a 5% and 17% increase in weight per unit area was observed for brick specimens incorporating 50% (M5) and 45% (M4) coal ash contents compared to coal ash bricks with 55% (M6) coal ash content. It may be attributable to the high specific gravity of cement (3.14) compared to coal ash (2.38). This increase in weight per unit area of the brick specimens incorporating cement contents of 15% and beyond (M3, M4, M5, and M6) was still comparable with burnt clay bricks. The results conform to previous research. Abbas et al. reported around an 18% reduction in weight per unit area of bricks incorporating 25% waste fly ash [42]. This was also noticeable in the results of previous research that the incorporation of porous material in clay or concrete masonry resulted in more lightweight bricks [43,44]. Moreover, around a 4% reduction in the unit weight of bricks incorporating 15% rice husk ash was also reported [43]. It can be observed that the incorporation of porous material in bricks leads to the development of more lightweight bricks [43,44,45].

#### 3.2.5. Water Absorption

**Figure 13:**
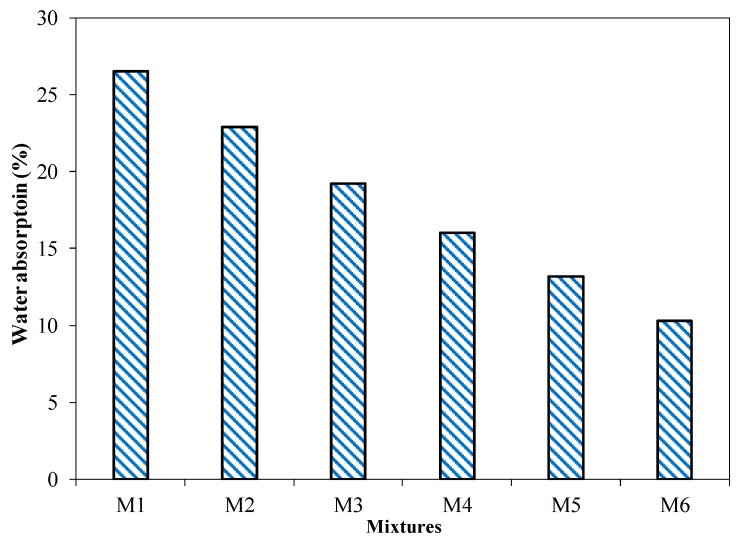
Water absorption of the brick specimens according to the percentage of cement.

Figure 13 shows the water absorption rates of brick specimens incorporating different coal ash and cement contents. The results of five specimens with a COV of less than 5% are presented at the 28-day mark. The water absorption of brick specimens incorporating 55 to 30% coal ash ranged from 26.5 to 10.3%, respectively. It was observed that the water absorption capacity of the brick specimen decreased with the decrease in coal ash content. Similar results have been reported in a previous study [46]. It can be observed that the water absorption of brick specimens incorporating different quantities of CA is in agreement with the mechanical properties of bricks. It should be noted that an increased percentage of coal ash in brick specimens has increased the water absorption capacity of the bricks, due to the water-absorbent nature of coal ash, which may exhibit the same characteristics as those of biomass ash [46,47,48]. Water absorption is primarily dependent on the distribution of pores available in the matrix [49]. Pores available in the coal ash may result in an increase in the water absorption of the matrix [42,46].

#### 3.2.6. Initial Rate of Absorption

**Figure 14:**
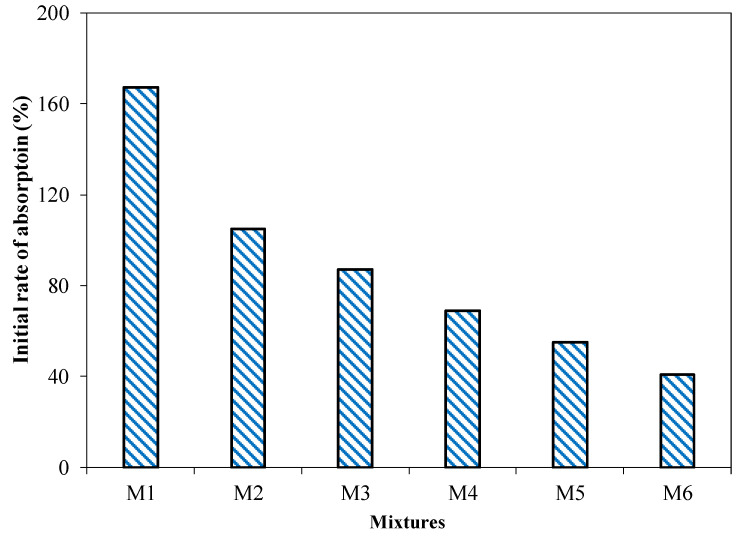
Water absorption performance of the brick specimens, with the percentage of cement.

The initial rate of absorption of bricks is one of the important factors for the bond between the bricks and the mortar. This bond between brick and mortar is affected by the water absorption value of bricks and the water-retaining capacity of mortar. The hydration water level for mortar may be reduced with the increased absorption capacity of bricks, leading to a weaker bond between brick and mortar. However, the lower initial rate of absorption (much lower absorption of water by bricks) can cause the mortar bed to float the brick course. Both scenarios will lead to a poor bond between bricks and mortar [50]. Hence, bricks with a higher initial rate of absorption should be wetted well before utilizing. The initial rate of absorption of brick specimens incorporating different percentages of cement and coal ash are shown in Figure 14. An average of five specimens with a COV of less than 5% at 28 days were reported. It was observed that the initial rate of absorption of the brick specimens increased with the increased amount of coal ash in the coal ash brick specimens. The initial rate of absorption of the brick specimens incorporating 55 to 30% coal ash ranged from 0.47 g/cm^2^/min to 0.12 g/cm^2^/min. For instance, a brick specimen incorporating 55% of coal ash showed an initial rate of absorption of 0.47 g/cm^2^/min in comparison to a brick specimen incorporating 30% coal ash exhibited 0.12 g/cm^2^/min. This may be attributed to the fact that the increased cement content in the coal ash brick specimen leads to the formation of calcium silicate hydrate, reducing the porosity of the coal ash specimens [46,47].

#### 3.2.7. Efflorescence and Appearance

**Figure 15:**
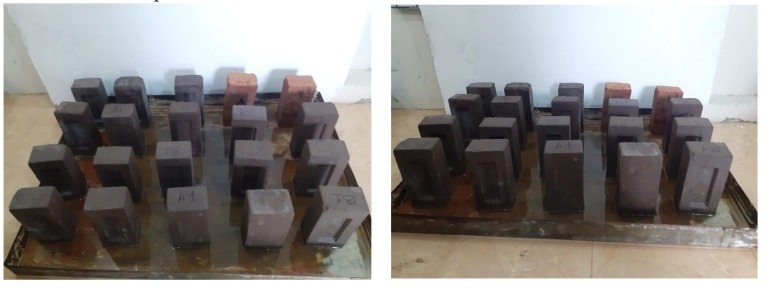
Efflorescence of the brick samples.

An esthetic problem was caused by the deposition of a thin, cloudy white salt on the brick surface, which is known as efflorescence [51]. Figure 15 shows a brick specimen exposed to efflorescence conditions, in accordance with ASTM C67. It was observed that the coal ash bricks showed no efflorescence, while slight efflorescence was observed on burnt clay specimens after 7 days, as per ASTM C67. Meanwhile, brick specimens were also evaluated for efflorescence after 90 days; it was observed that the burnt clay bricks showed minor efflorescence (10% of the surface area) in comparison to that of the coal ash bricks, which showed slight efflorescence (5% of the surface area). Brick specimens incorporating a higher percentage of coal ash showed lesser efflorescence, which may be attributed to the density of particles at the micro-level. Similar findings have previously been reported elsewhere [52]. The reduction in efflorescence in the coal ash compressed bricks may be attributed to achieving better particle density, leading to reduced porosity. It should be noted that reduced porosity leads to the lower transportation of salts through the capillary pores, resulting in a reduction in efflorescence [53,54]. Generally, ferric oxide, Fe_2_O_3_, can also play a role in efflorescence; normally, a less than 10% value of Fe_2_O_3_ is recommended for efflorescence control [55]. Therefore, almost no efflorescence was observed in the brick specimens incorporating coal ash and cement as per ASTM C67.

**Figure 16:**
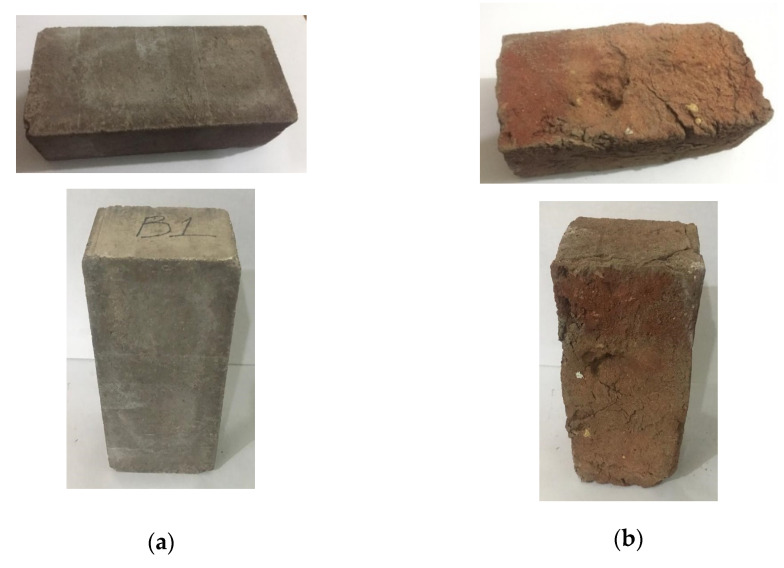
Surface appearance (**a**) coal ash brick (**b**) burnt clay brick.

Figure 16 shows the appearance of coal ash and burnt clay bricks. It can be observed that the coal ash bricks showed a smooth and uniform finish in comparison to that of burnt clay bricks. This uniform, smooth finish on the coal ash bricks may be attributed to the compressible molding and normal curing without firing, whereas in the case of burnt clay bricks, it is evident that there are rough surfaces and variations in texture on the brick faces. Rough surfaces and uneven finish may lead to uneconomical structural finishes and repairs.

#### 3.2.8. XRD Analysis

**Figure 17:**
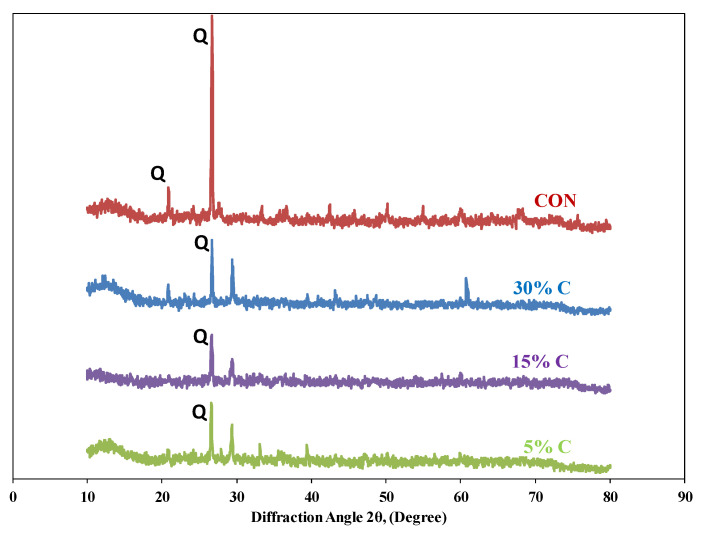
XRD of the brick specimens with different percentages of cement. (CON represents the control specimen).

The different phases of chemicals in the brick specimens were investigated via X-ray diffraction analysis. Figure 17 shows the results of XRD for burnt clay and coal ash brick specimens incorporating different quantities of cement. The peaks of quartz (Q) and calcite (C) were identified in both the burnt clay specimens and coal ash-based brick specimens incorporating different cement contents. Minerals such as quartz, anhydrite, and mullite have been identified in previous studies [56,57,58,59]. The presence of calcite (C) is also confirmed in the XRD analysis, which was also reported in a previous study [60].

#### 3.2.9. Relationship between Mechanical Properties and the Durability of Bricks

**Figure 18:**
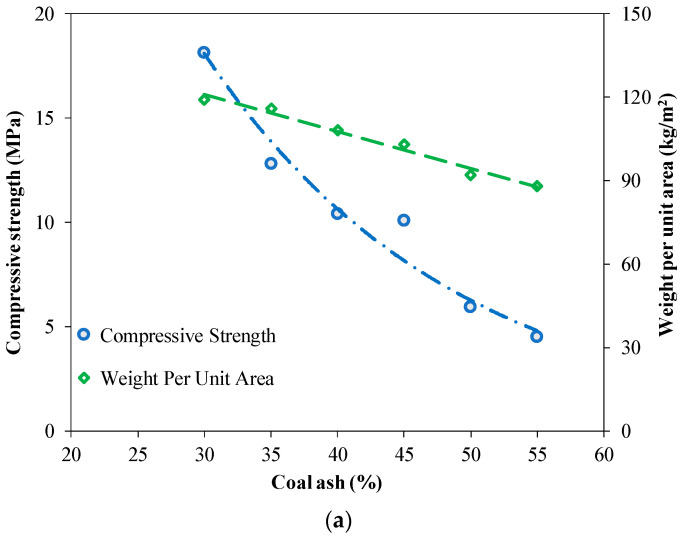

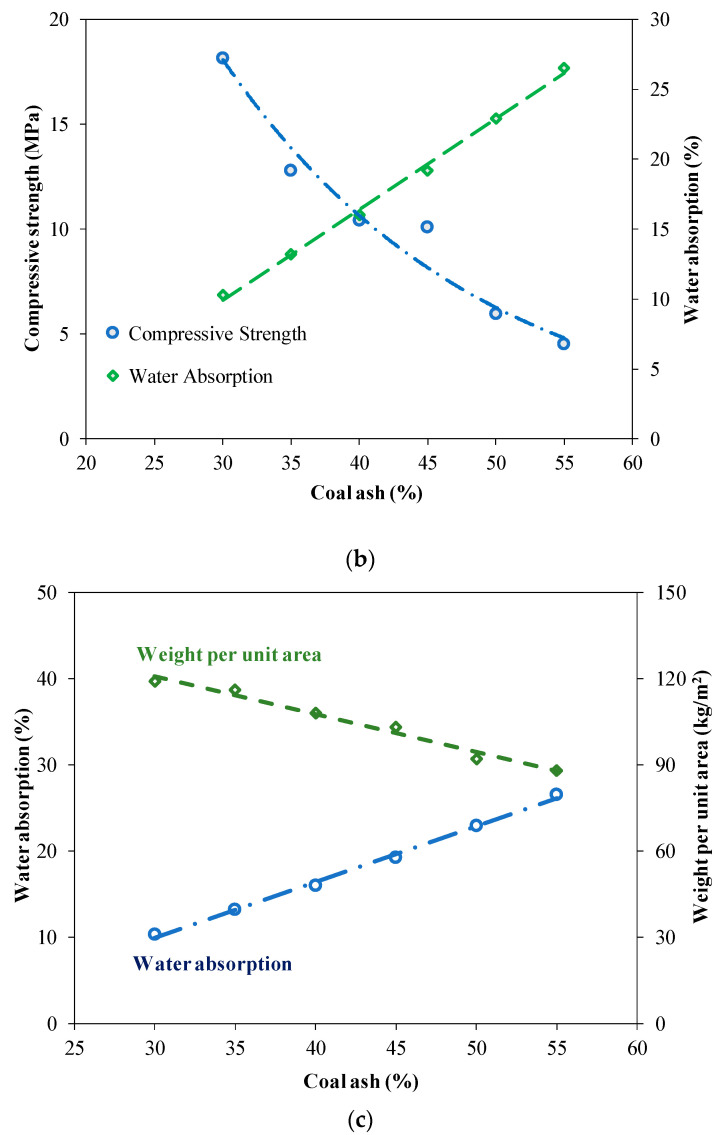
Relationship between the mechanical properties and the durability of bricks. (**a**) Relationship between compressive strength and weight per unit area, (**b**) Relationship between compressive strength and water absorption, (**c**) Relationship between water absorption and weight per unit area.

The relationship between the physical and mechanical properties of bricks was established, as shown in Figure 18. Figure 18a depicts the relationship between compressive strength and weight per unit area of bricks incorporating different quantities of coal ash. It is quite evident that the compressive strength decreases with the decrease in weight per unit area, due to the more porous structure of hydrated products at the micro-level [61]. Similarly, the relationship between compressive strength and the water absorption of bricks incorporating different percentages of coal ash was established, as shown in Figure 18b. It can be seen that the compressive strength of the bricks decreased with an increase in water absorption. The increase in water absorption of the brick specimens due to the increased content of coal ash may be attributed to the hydrophilic nature of coal ash, ultimately leading to a reduction in compressive strength [62].

Figure 18c presents the relationship between water absorption and weight per unit area of the brick specimens incorporating different contents of coal ash. An inverse relationship between water absorption and weight per unit area has been observed. It has been suggested that weight per unit area decreases and water absorption increases with the increased content of coal ash. The results presented in Figure 18 showed that a coal ash content of up to 45% is in the allowable range, in accordance with ASTM C62 limits.

#### 3.2.10. Cost Analysis

**Figure 19:**
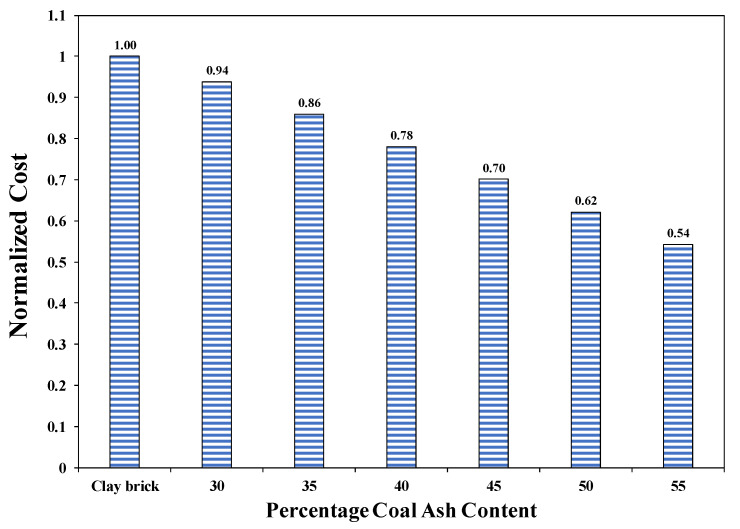
Cost Comparison of Coal Ash and Burnt Clay brick.

The commercial utilization and choice of coal ash bricks in the construction industry are mainly dependent on their cost effectiveness, while meeting the criteria of ASTM and building codes. Hence, the cost of manufactured coal ash bricks was calculated using the market rate system (MRS) of Punjab [63] for the mixtures mentioned in Table 2. The market rate for cement was 610 Pakistani rupees (PKR) (USD 3.2) for one bag (50 kg) and the rate for sand was PKR 40 (USD 0.21) per cubic ft. Meanwhile, the cost of coal ash was PKR 4000 (USD 21) for one thousand kg (1 ton). The cost of electrical energy per brick is PKR 0.45 (USD 0.002). The availability of local materials at cheaper rates leads to obvious economic benefits. The validation of the cost analysis was also verified by the recent research works published by another researcher [62]. Hence, the normalized cost for different coal ash bricks is shown in Figure 19. Results revealed that the cost of manufacturing the bricks was reduced with the increased amount of coal ash content. For instance, about 30% of the cost can be reduced by utilizing 45% coal ash in the production of bricks meeting ASTM limits and Pakistan Building Code requirements. For coal ash bricks incorporating coal ash from 5% to 55%, the cost varies from 46% to 6%, respectively. Hence, cost analysis suggests that the utilization of coal ash in the production of bricks leads to economical, low-cost, and sustainable building units.

#### 3.2.11. Microstructural Analysis

**Figure 20:**
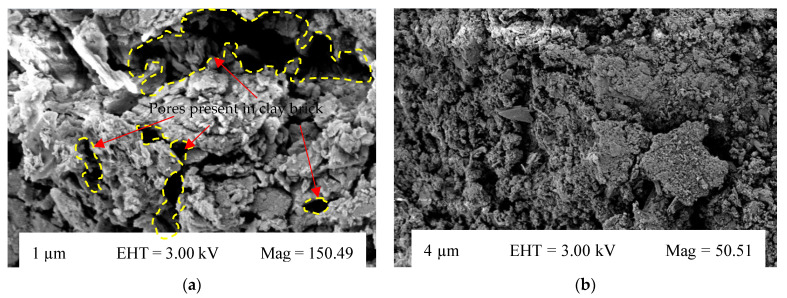

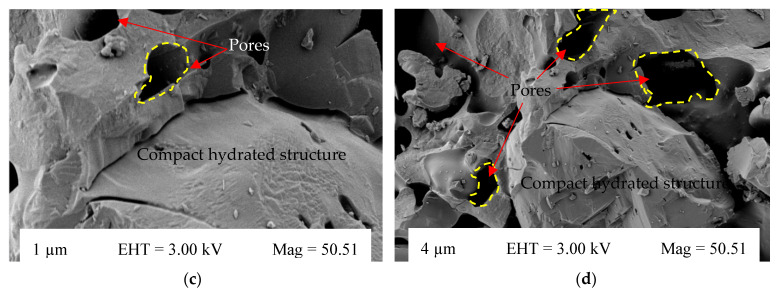
Scanning electron microscopy (SEM). (**a**) SEM of a burnt clay brick, (**b**) SEM of a burnt clay brick, (**c**) SEM of a coal ash brick, (**d**) SEM of a coal ash brick.

Figure 20a–d shows the high-resolution, magnified scanning electron microscopy (SEM) images of burnt clay bricks and coal ash bricks at different magnifications. It can be observed that the microstructure of bricks incorporating coal ash was porous, which may be attributed to the porous nature of coal ash [61]. This may be the possible reason for their lesser weight per unit area than conventional burnt clay bricks. Conventional burnt clay bricks showed a comparatively uniform and homogeneous microstructure. Similarly, the bricks incorporating coal ash showed a compacted hydrated structure, along with pores that were irregular and interconnected. These pores that are present in the coal ash brick specimens may be related to the process of crystallization [64]. The reduction in compressive strength with an increase in coal ash percentage can also be supported by the SEM images. Furthermore, the water absorption and weight per unit area results are also consistent with the microstructural analysis of the brick specimen.

## 4. Conclusions

In the current study, the potential of coal ash generated through thermal coal power plants for the production of unburnt compressible bricks was studied. The utilization of locally available coal ash as a raw material for the production of unburnt compressible brick can be seen as a viable option for the recycling of the abundant waste generated by power plants. The higher content of coal ash makes the bricks a low-cost material, which can react and contribute to the development of sustainable brick production. The following conclusions were drawn from the results of the current study:

1. Coal ash and cement can be used in the production of sustainable coal ash bricks. The results revealed that coal ash up to 45% in combination with cement can be used for the production of coal ash bricks, to be used in the construction industry. The compressive and flexural strengths of coal ash brick decreased with the increased proportions of coal ash. However, coal ash bricks incorporating 45% coal ash in combination with 15% cement showed a compressive strength of 10 MPa, satisfying the minimum specified compressive strength required by the Building Code of Pakistan for masonry construction. At the same time, all the brick specimens incorporating coal ash satisfied the criteria of the minimum modulus of rupture, in accordance with ASTM C67 (i.e., >0.65 MPa).
2. It was evident from the results that the overall water absorption and the initial rate of absorption increased with the increased quantities of coal ash used in coal ash brick production. Bricks incorporating 30 to 55% of coal ash showed a water absorption of 10.3 to 26%; therefore, coal ash bricks can be used in moderate weathering conditions. All the coal ash brick specimens exhibited a higher initial rate of absorption. The tested brick specimens showed an initial rate of absorption greater than 0.1 g/min/cm^2^; hence, coal ash bricks should be submerged in water before their utilization in construction. The increased percentage of coal ash may lead to lighter-weight bricks, reducing the overall structure’s weight. Furthermore, it was evident from the results that the efflorescence resistance was significantly enhanced with the incorporation of coal ash.
3. It can be concluded from the results of the current study that up to 45% of coal ash in the presence of cement can be used for the production of coal ash bricks, and can be used in the industrial-scale production of bricks for sustainable and economical construction. Furthermore, the feasibility of replacing the coal ash with other ingredients for coal ash bricks, such as quarry dust and sand, provides an interesting outcome that warrants future detailed study.

## Figures and Tables

**Table 1:** Chemical compositions of coal ash, cement, sand, quarry dust, and clay.

Constituents (%)	Coal Ash	Cement	Sand	Quarry Dust	Clay
CaO	6.08	61.47	1.25	42.84	8.61
MgO	0.83	2.68	0.32	2	2.46
SiO_2_	61.5	19.06	91.7	9.7	58.1
SO_3_	4.31	2.54	0.23	2.18	-
Al_2_O_3_	10.29	5.68	0.95	1.72	12.01
Fe_2_O_3_	4.42	4.34	0.67	0.81	5.02
K_2_O		0.71			2.34
Na_2_O		0.28			1.91
Na_2_O_e_					
LOI	10.39	3.24	1.94	38.41	9.46
Specific Gravity	2.38	3.14	2.61	2.53	2.23

**Table 2:** Mixtures for the development of bricks.

Materials	M1	M2	M3	M4	M5	M6
Coal ash (%)	55	50	45	40	35	30
Cement (%)	5	10	15	20	25	30
Sand (%)	30	30	30	30	30	30
Quarry dust (%)	10	10	10	10	10	10

**Table 3:** Test results and coefficient of variation (COV) for different mixtures.

Tests		Values, (COV, %)				
	M1	M2	M3	M4	M5	M6
Weight per unit area (kg/m^2^)	88.3(1.26)	92.2 (0.95)	103.1 (0.75)	108.2 (0.32)	116.3 (1.05)	118.1 (0.85)
Compressive strength (MPa)	4.52 (1.33)	5.95 (1.43)	10.1 (2.01)	10.4 (0.85)	12.8 (3.47)	18.15 (1.02)
Flexural strength (MPa)	1.01 (0.98)	1.05 (1.08)	1.38 (2.36)	1.95 (1.42)	2.56 (2.81)	3.47 (0.32)
Water absorption (%)	26.5 (0.15)	22.9 (0.39)	19.2 (0.84)	16 (0.55)	13.2 (1.02)	10.3 (1.04)

**Table 4:** The properties specified by the different standards.

Standard	Description	Compressive Strength (MPa)	Bulk Density (kg/m^3^)	Water Absorption (%)
ASTM C62	Severe Weathering	20.7	-------	-------
	Moderate Weathering	17.2	-------	-------
	Negligible Weathering	10.3	-------	-------
ASTM C55	Normal Weight	13.8	2000	10
Pakistan Building Code		8	----	-----
Chinese National Standard	First-Class Brick	15	1800–2000	15 (Maximum)
	Second-Class Brick	9.8	1800–2000	19 (Maximum)
Indian Standard	First-Class Brick	5–10 (Load Bearing)	-------	15 (Maximum)
	Second-Class Brick	3–5 (Non-load Bearing)	-------	20 (Maximum)
Brazilian Standard	------	1.5 (Minimum)	------	------

## Data Availability

The data presented in this study are available upon request from the corresponding author. The data are not publicly available due to privacy.
